# Childhood Smoking, Adult Cessation, and Cardiovascular Mortality: Prospective Study of 390 000 US Adults

**DOI:** 10.1161/JAHA.120.018431

**Published:** 2020-10-28

**Authors:** Blake Thomson, Jonathan Emberson, Ben Lacey, Richard Peto, Mark Woodward, Sarah Lewington

**Affiliations:** ^1^ Nuffield Department of Population Health (NDPH) University of Oxford United Kingdom; ^2^ The George Institute for Global Health University of Oxford United Kingdom; ^3^ MRC Population Health Research Unit NDPH University of Oxford United Kingdom; ^4^ The George Institute for Global Health University of New South Wales Sydney Australia; ^5^ Department of Epidemiology Johns Hopkins University Baltimore MD

**Keywords:** Risk Factors, Lifestyle, Epidemiology

Smoking still causes about 100 000 US deaths per year from cardiovascular disease.^1^ There were an estimated 25 million daily smokers in the United States in 2018, including 5 million who began smoking regularly in childhood (defined here as before age 15 years); of these, an estimated 0.5 million began before age 10 years.^2^ Previous research has shown that, among daily smokers, those who began youngest have the greatest relative increase in overall adult mortality,^2^ but better evidence is needed on the relevance of the age when smoking began to cardiovascular adult mortality. We investigated this using the annual US National Health Interview Surveys (a cross‐sectional household interview survey of the noninstitutionalized US population) linked to the National Death Index.^3^ Further details about the National Health Interview Surveys, and the anonymized data that support the findings of this study, are publicly available online (https://www.cdc.gov/nchs/nhis/data‐questionnaires‐documentation.htm).

In 1997 to 2014, 424 793 US adults aged 25 to 74 years were surveyed and have been followed for mortality to December 31, 2015. Participants were asked about their health, habits (including smoking, drinking, and physical activity), medical history, and demographic details. Current smokers were categorized by the age they began smoking regularly (as reported at recruitment): <10, 10 to 14, 15 to 17, 18 to 20, or >20 years. Occasional smokers were excluded. Ex‐smokers were categorized by the age they last smoked regularly: 15 to 34, 35 to 44, 45 to 54, or 55 to 64 years (excluding the few who had quit at older ages). Ex‐smokers who quit within 5 years of death were excluded to reduce reverse causality (by which smokers quit because of the onset of serious illness). Causes of death were recorded according to the *International Classification of Diseases, Tenth Revision* (*ICD‐10*); our analyses of cardiovascular mortality included cardiac (I00–I09, I11, I13, I20–I51) and cerebrovascular (I60–I69) causes. Prospective analyses were restricted to “premature” deaths (here defined as ages 25–74 years).

Cox regression was used to estimate mortality rate ratios (RRs) comparing different smoking histories. For each baseline‐defined smoking group (including the reference group with RR = 1.0), the RR is shown with a group‐specific 95% CI that reflects the variance of the log risk in just that 1 group.^4^ (The variance of the log RR comparing 2 different groups is the sum of the variance of the log risk in 1 group and the variance of the log risk in the other group.) The RRs were adjusted for age‐at‐risk (in 5‐year groups; 10 categories), sex, race (5 categories), education (4 categories), region (4 categories), and alcohol consumption (5 categories). All participants provided informed consent. Ethics approval for analyses of this publicly available anonymized dataset was not needed. Analyses used SAS v.9.4 and R v.3.1.1.

After excluding participants with missing information on smoking, covariates of interest, or mortality linkage, there were 390 929 participants aged 25 to 74 years (mean age 47, 56% female) at recruitment: 228 165 (58%) never smokers, 88 717 (23%) ex‐smokers, and 74 047 (19%) current smokers. Among current smokers, 1403 (2%) had begun before age 10 and 14 421 (19%) at ages 10 to 14 years. During 3.5 million person‐years of follow‐up, 4479 died of cardiovascular disease before age 75: 1579 never smokers, 1227 ex‐smokers, and 1673 current smokers. For current smokers (Figure), the adjusted cardiovascular mortality RRs associated with starting to smoke regularly at ages <10, 10 to 14, 15 to 17, 18 to 20, and >20 years were 4.89 (95% CI, 3.90–6.12), 2.98 (2.68–3.31), 2.87 (2.64–3.13), 2.66 (2.41–2.94), and 2.45 (2.18–2.75), respectively, as against 1.00 (0.95–1.06) in never smokers. Comparing current smokers who began in childhood (age <15 years) versus later (age ≥15 years), the RRs were 3.20 (2.90–3.52) and 2.69 (2.54–2.85), respectively, compared with never smokers (1.00; 0.95–1.06). For ex‐smokers (Figure), the RRs associated with smoking cessation at ages 15 to 34, 35 to 44, 45 to 54, or 55 to 64 years were 0.91 (0.81–1.02), 1.19 (1.06–1.33), 1.58 (1.42–1.76), and 1.69 (1.47–1.93), as against 2.80 (2.66–2.95) in current smokers and 1.00 (0.95–1.06) in never‐smokers.

Overall, current smokers in this contemporary US population had nearly 3 times the risk of premature cardiovascular mortality compared with never smokers. The risk was higher among those who had begun smoking in childhood (<15 years), and highest of all for those who had begun before age 10 years. However, quitting at any age was associated with a substantially lower risk than continuing to smoke, with the greatest risk reduction among those who quit before age 40 years.

Age at starting to smoke is an important, but underappreciated, determinant of adult cardiovascular mortality, and this study indicates that the ≈5 million US smokers who began before age 15 years are at especially high risk of premature death from cardiovascular disease if they do not quit. If the associations between smoking and cardiovascular mortality are largely causal, then smoking is a cause of more than two thirds of all premature deaths from cardiovascular disease among smokers who began before age 15 years. However, smoking cessation substantially reduced the excess risk of cardiovascular death, with those who quit before age 40 years (preferably well before age 40 years) avoiding >90% of the excess cardiovascular risk, as well as avoiding substantial excess risks of death from other tobacco‐associated diseases.^5^

## Sources of Funding

Thomson received scholarship support from the Nuffield Department of Population Health, University of Oxford. Emberson received grant support from the Medical Research Council (UK) during the conduct of the study. Lacey acknowledges support from the National Institute for Health Research Biomedical Research Centre (Oxford, UK) and the British Heart Foundation Centre of Research Excellence (Oxford, UK). Lewington received grant support from the Medical Research Council (UK) during the conduct of the study, and research funding from the US Centers for Disease Control and Prevention Foundation (with support from Amgen) outside the submitted work. The sponsors had no role in the study design, data collection, data analysis, data interpretation, or writing of the report.

## Disclosures

Emberson reports grant support from Boehringer Ingelheim outside the submitted work. Woodward has received consulting fees from Amgen, Inc. and grant support from the National Health and Medical Research Council of Australia. The remaining authors have no disclosures to report.

**Figure 1:**
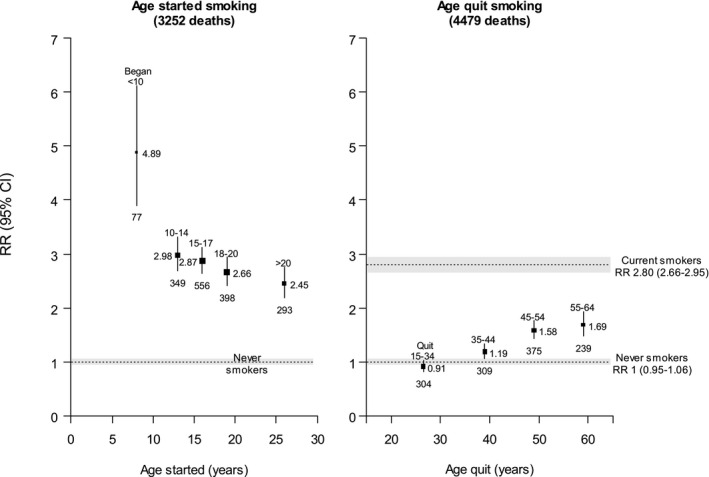
Cardiovascular mortality RRs by age started smoking among current smokers, and age quit smoking among ex‐smokers, compared with never smokers, age at risk 25 to 74 years. Shaded areas and error bars indicate 95% CI. Box area is inversely proportional to the variance of the log risk. Number below box indicates deaths in that category. NHIS data include waves 1997–2014 followed through December 31, 2015. Adjusted for age at risk, sex, education, alcohol consumption, race, and region. NHIS indicates National Health Interview Surveys; and RR, rate ratio.
